# Does Vibration Foam Roller Influence Performance and Recovery? A Systematic Review and Meta-analysis

**DOI:** 10.1186/s40798-022-00421-2

**Published:** 2022-03-04

**Authors:** Alejandra Alonso-Calvete, Miguel Lorenzo-Martínez, Alexis Padrón-Cabo, Alexandra Pérez-Ferreirós, Anton Kalén, Cristian Abelairas-Gómez, Ezequiel Rey

**Affiliations:** 1 REMOSS Research Group, Facultade de Ciencias da Educación e do Deporte, Universidade de Vigo, Pontevedra, Spain; 2 Department of Physical Education and Sport Science, Faculty of Sports Sciences and Physical Education, Campus Bastiagueiro, University of A Coruña, 15071 A Coruña, Spain; 3 CLINURSID Research Group, Psychiatry, Radiology, Public Health, Nursing and Medicine Department, Universidade de Santiago de Compostela, Santiago de Compostela, Spain; 4 Faculty of Education Sciences, Universidade de Santiago de Compostela, Santiago de Compostela, Spain; 5 Simulation and Intensive Care Unit of Santiago (SICRUS) Reseach Group, Health Research Institute of Santiago, University Hospital of Santiago de Compostela-CHUS, Santiago de Compostela, Spain; 6 School of Informatics, University of Skövde, Skövde, Sweden

**Keywords:** Foam rolling, Recovery modalities, Muscle adaptations

## Abstract

**Background:**

Foam rolling has been extensively investigated, showing benefits in performance and recovery. Recently, vibration has been added to foam rollers, with hypothesized advantages over conventional foam rollers. However, there is no systematic evidence in this regard.

**Objective:**

To carry out a systematic review and meta-analysis about the effects of vibration foam roller (VFR) on performance and recovery.

**Methods:**

A systematic search was conducted in PubMed/MEDLINE, Web of Science and SportDiscus according to the PRISMA guidelines. The outcomes included performance (jump, agility and strength) and recovery variables (blood flow, pain and fatigue) measured after an intervention with VFR. The methodological quality was assessed with the PEDro scale. A random-effects model was used to perform the meta-analysis.

**Results:**

Initially, 556 studies were found and after the eligibility criteria 10 studies were included in the systematic review and 9 in the meta-analysis. There was no significant effects on jump performance (SMD = 0.14 [95% CI − 0.022 to 0.307]; *p* = 0.101; *I*^*2*^ = 1.08%) and no significant beneficial effects were reported on isokinetic strength (SMD = 0.16 [95% CI − 0.041 to 0.367]; *p* = 0.117; *I*^*2*^ = 9.7%). Recovery appears to be enhanced after VFR interventions, but agility does not seem to increase after VFR interventions.

**Conclusion:**

This systematic review and meta-analysis suggest that VFR could have great potential for increasing jump performance, agility, strength and enhancing recovery. Further research is needed to confirm the effects of VFR on performance and recovery.

*Trial Registration* This investigation was registered in PROSPERO with the code CRD42021238104.

## Key Points

- Vibration foam roller is suggested as an effective tool to increase jump performance and recovery.
- The potential benefits of vibration foam roller on agility and strength need to be confirmed with further investigation.
- The underlying physiological effects of vibration foam rollers are unclear.

## Background

Massage rollers have been described as an effective method to decrease thickening, adhesion and, the tension of the fascial tissue and muscles [1, 2]. These effects could be achieved with many devices, but in recent years one of the most widely used is the foam roller (FR) [3, 4]. During foam rolling, soft tissues are rolled and compressed by applying bodyweight, which has been demonstrated to stimulate the muscle and fascial tissue, generating changes at neuromuscular level [4, 5]. The benefits of FR have been largely described, showing an increase of range of motion (ROM) [3], decrease in pain [4, 6] and effects on performance and recovery [7]. In addition, FR has become a popular practice before and after different sports, due to its affordability, ease, and time-efficient applicability [4].

Recently, vibration has been added to the FR devices with the aim of increasing their benefits [8]. Specifically, this vibration was expected to produce an in-depth stimulation of the tissues rolled, especially the mechanoreceptors of the joints and blood vessels [9, 10]. Moreover, a greater contribution of the mechanoreceptors has been reported with vibration foam roller (VFR), suggesting that the vibration could influence deeper into the tissue through mechanisms of the neuromuscular system but also at a central level [8, 11–13]. In this regard, VFR appears to have higher benefits than FR in ROM [14–17], performance [7, 18, 19] and recovery [9, 10, 20], but more evidence is needed to support these results since several studies also reported detrimental effects in jump and strength performance [15, 21]. FR and VFR are easy to use and their benefits could be achieved with short-time interventions [7, 21]. For these reasons, the use of these tools has been included in several sport practices, both in the warm-up and after exercise to cool down or decrease the effects of exercise-induced fatigue [9–11]. Nevertheless, VFR has been less studied, and it is considerably more expensive than FR, so its value is still unclear.

To date, several systematic reviews have been conducted to analyze the effects of FR on recovery and performance [4, 6, 7, 11, 21]. Overall, these studies showed positive effects of FR on performance both pre and post-exercise and on recovery, analyzing fatigue and pain after exercise [22]. However, despite the increasing scientific interest in VFR and its benefits, the evidence is controversial and there is still no consensus about this tool. Nevertheless, considering the prior neurophysiological explanations, VFR appears to have great potential [23]. Moreover, it has to be pointed out that to date no systematic reviews or meta-analysis examining the effects of VFR on these variables have been conducted. Bearing in mind the aforementioned considerations, this study aimed to carry out a systematic review and meta-analysis about the acute effects of VFR on performance and recovery.

The PICO (Population, Intervention, Comparison, Outcomes) question was as follows: Does vibration foam roller (I) influence performance and recovery (O) prior or after exercise (C) in healthy subjects (P)?

## Methods

A systematic review with meta-analysis was carried out according to the Preferred Reporting Items for Systematic Reviews and Meta-Analyses (PRISMA) guidelines [24] and following the recommendations for Wager and Wiffen for ethical publishing of systematic reviews [25]. This investigation was registered in PROSPERO with the code CRD42021238104.

### Search Strategy

In April 2021, a systematic and structured literature search was conducted in PubMed/MEDLINE, Web of Science and SportDiscus. The terms for each search were similar and according to the requirements of each database, using the free text words: “vibration foam roller”, “vibration foam rolling”, “vibration rolling” and “vibration roller” linked by “OR”.

### Inclusion and Exclusion Criteria

The selection criteria were established according to the PICO question as follows:

- *Participants* Studies with subjects aged ≥ 18 years old were included in this systematic review and meta-analysis. Those who did not show information about age were discarded. In addition, investigations with healthy subjects were selected and those with chronic or acute injuries were considered ineligible.
- *Intervention* All articles included performed at least one intervention with VFR. Studies with interventions based only on foam roller without vibration or roller massages were discarded.
- *Comparison* Studies comparing VFR interventions with other methods such as foam roller without vibration, stretching, rest or massage were selected for this review.
- *Outcomes* The outcomes selected were recovery and performance variables.
- *Type of Study* Original articles with at least one intervention based on VFR and published in English or Spanish were included.
- *Exclusion Criteria* Studies with no intervention or performed on unhealthy people were excluded. Additionally, letters to the editor, systematic reviews and meta-analysis, abstracts, opinion or conference papers were also excluded.

### Study Selection and Data Extraction

Two independent investigators (A.A.C and A.P.F) performed the screening, eligibility and extraction of the data from the studies in order to avoid potential bias. In case of disagreement, a third investigator (A.K) was consulted to reach a decision. All this process was performed based on the minimum requirements of Cochrane for Inclusion and Exclusion Criteria [26].

### Methodological Quality

All the studies included were evaluated using of the PEDro scale, which has been demonstrated to be a reliable and valid method to assess the methodological quality of intervention studies [27]. This scale presents 11 items in three different sections: eligibility and randomization of the subjects (1–4), blinding (5–7) and consistency of the results (8–11). The score for each study was calculated by summing the score of 10 items examining the potential sources of bias, giving one point to the study if it clearly satisfies the criteria. Two investigators performed independently the evaluation of the studies (A.A.C and A.P.F) and after discussion, a third investigator was consulted in case of disagreement (A.K).

### Statistical Analysis

The statistical analysis was performed using Comprehensive Meta-Analysis software version 2.2.064 for Windows (Biostat Inc., Englewood, New Jersey, United States). Random effects models were conducted to determine and compare the effects between pre-and post-intervention of VFR on the jump and isokinetic strength performance. The standard mean difference (SMD) values with 95% confidence intervals were used to estimate the magnitude of foam roller vibration intervention. The SMD were interpreted as trivial (SMD < 0.2), small (0.2 ≥ SMD < 0.5), moderate (0.5 ≥ SMD < 0.8), or large (SMD ≥ 0.8) [28]. The significance level was established at *p* < 0.05. Heterogeneity was evaluated using the *I*^*2*^ statistic. This statistic represents the percentage of variation in estimated effects across studies due to heterogeneity rather than chance. According to Higgins et al. [26] the *I*^2^ was interpreted as low (*I*^2^ < 25%), moderate (25% ≥ *I*^2^ < 75%), and high (*I*^2^ ≥ 75%).

## Results

### Study Selection

**Fig. 1:**
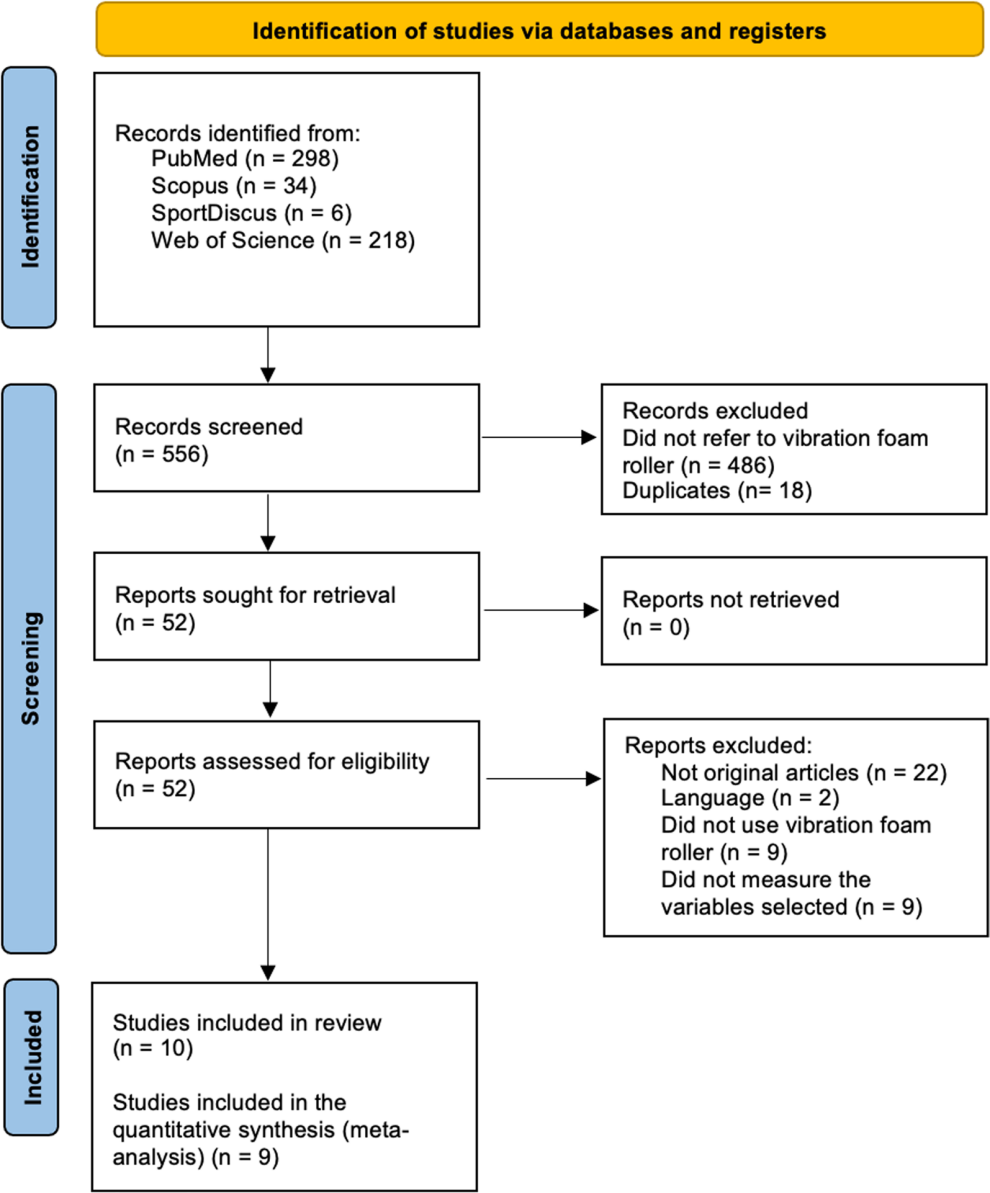
Flow chart outlining the search process

The initial systematic search retrieved 556 articles and after applying the inclusion and exclusion criteria and removing duplicate items, a total of 10 studies were finally included for the systematic review and 9 of them for the quantitative synthesis with meta-analysis. The process of the systematic search is described in Fig. 1 through a flow chart according to PRISMA guidelines.

Initially, 11 studies were selected but one [29] was excluded as it did not meet all the inclusion criteria. Specifically, this study appears to assess joint performance, but an in-depth analysis showed that the variable examined was the range of movement, which is not included in the eligibility criteria of this systematic review and meta-analysis. Moreover, one study [19] was excluded from the meta-analysis since the design of the investigation did not provide pre-test measurements and therefore, the meta-analysis could not be conducted.

### Characteristics of the Studies

The main characteristics of the 10 studies included in this systematic review are shown in Table 1. Jointly, they evaluated a total of 236 subjects (74.1% male) through a crossover design (*n* = 7) [9, 13, 14, 19, 29–31] or a randomized trial (*n* = 3) [10, 15, 18]. Seven investigations [9, 10, 13–15, 29, 31] compared the use of VFR with FR and other studies analyzed the effects of VFR in comparison with static (*n* = 4) [14, 19, 27, 28] or dynamic stretching (*n* = 3) [15, 19, 27]. The main outcomes analyzed in performance were jump (*n* = 6) [10, 13, 15, 18, 29, 31], agility (*n* = 3) [18, 27, 28] and isokinetic strength (*n* = 4) [14, 19, 30, 31]. Additionally, in order to assess the recovery, the studies included measurements of blood flow [9], fatigue [19] and pain [10].

**Table 1:** Summary of the characteristics of the studies and results of the intervention

Study	Study population	Muscles involved	Intervention	Frequency/time	Outcome	Results
Chen et al. [19]	Handball players (female = 10) 21 ± 1 years	Quadriceps and hamstrings	Crossover 1: SS + DS 2: DS 3: DS + VFR	45 Hz/8 min	Isokinetic strength and fatigue recovery (Thorstensson test)	No differences in strength Fatigue decreases after DS + VFR
Hsu et al. [30]	23 elite table tennis players (female = 9; male = 14) 20.6 ± 0.8 years	Gastrocnemius, quadriceps, hamstrings, low back, and rotator cuff	Crossover 1: DS + SS 2: DS + FR 3: DS + VFR	33 Hz/1 min	Jump performance (Board jump test) and agility (Edgren Side Step Test)	DS + FR and DS + VFR increase agility similarly Jump performance increase similarly with all interventions
Lai et al. [9]	23 runners (female = 11; male = 12) 26.4 ± 6.5 years	Gastrocnemius	Crossover 1: FR 2: VFR	20–40 Hz/6 min	Recovery (blood flow)	Blood flow increases similarly with both methods
Lim et al. [15]	20 healthy subjects (female = 6; male = 14) 20.97 ± 1.56 years	Hamstrings	Randomized trial 1: FR 2: VFR	32 Hz/10 min	Jump performance (Vertical jump test)	No differences in jump performance with any intervention
Lin et al. [18]	40 badminton players (female = 15; male = 25) 21.4 ± 1.5 years	Gastrocnemius, hamstrings, quadriceps, rotator cuff and low back	Randomized trial 1: DS 2: DS + VFR	28 Hz/20 s	Jump performance (CMJ) and agility (FITLIGHT test)	Jump performance and agility improve similarly with both interventions
Romero-Moraleda et al. [10]	38 healthy subjects (female = 6; male = 32) 22.2 ± 3.2 years	Vastus lateralis, vastus medialis and rectus femoris	Randomized trial 1: FR 2: VFR	18 Hz/5 min	Recovery (PPT and VAS) and jump performance (CMJ)	Pain perception decreases more with VFR than with FR. Both improved similarly PPT and jump performance
Tsai et al. [13]	Volleyball players (male = 16) 21.5 ± 1.15 years	Quadriceps, gluteus, biceps femoris, tibialis anterioris, gastrocnemius, iliotibial band and plantar fascia	Crossover 1: FR 2: VFR 3: rest	45 Hz/15 min	Jump performance (Drop jump test)	FR increase jump performance and VFR does not increase jump performance
Lyu et al. [31]	Healthy subjects (male = 20) 21 ± 1.01 years	Gastrocnemius	Crossover 1: VFR 2: VFR + DC 3: SS	28 Hz/3 min	Isokinetic muscle strength and agility (figure-of-8 hop test)	VFR and VFR + DC increase similarly muscle strength and agility
Lee et al. [14]	Healthy subjects (male = 30) 20.4 ± 1.2 years	Quadriceps and hamstrings	Crossover 1:VFR 2: FR 3: SS	28 Hz/6 min	Isokinetic muscle strength	VFR increase isokinetic strength in quadriceps and hamstrings more than SS but similar to FR
Nakamura et al. [21]	Healthy subjects (16 = male) 21.7 ± 1.3 years	Plantar flexors	Crossover 1: VFR 2: FR 3: rest	48 Hz/4 min	Isokinetic strength and jump performance (Drop jump test)	VFR does not increase isokinetic strength or jump performance

SS: static stretching; DS: dynamic stretching; DC: dynamic contraction; FR: foam roller; VFR: vibration foam roller; CMJ: counter movement jump; PPT: pressure pain threshold; VAS: visual analogue scale

### Methodological Quality

All the studies included were analyzed in terms of methodological quality with the PEDro scale [27]. Table 2 describes the score in each study for each item and the total score obtained, finding nine articles with a score of 5/11 [9, 10, 13–15, 18, 29–31] and one with 3/11 [19]. As shown in Table 2, criteria 3, 5 and 6 were not satisfied in any of the included studies.

**Table 2:** Results of the methodological quality evaluation using the PEDro scale

	Romero-Moraleda et al. [10]	Hsu et al. [30]	Lim and Park [15]	Tsai et al. [13]	Chen et al. [19]	Lin et al. [18]	Lai et al. [9]	Lyu et al. [31]	Lee et al. [14]	Nakamura et al. [21]
Inclusion criteria	−	+	+	+	−	+	+	+	+	+
Random allocation	+	+	+	+	+	+	+	+	+	+
Concealed allocation	−	−	−	−	−	−	−	−	−	−
Similarity at baseline	+	+	+	+	−	+	+	+	+	+
Subject blinding	−	−	−	−	−	−	−	−	−	−
Therapist blinding	−	−	−	−	−	−	−	−	−	−
Assessor blinding	+	−	−	−	−	−	−	−	−	−
> 85% follow-up	−	−	−	−	−	−	−	+	−	−
Intention to treat	−	−	−	−	−	−	−	+	−	−
Between-group comparison	+	+	+	+	+	+	+	+	+	+
Point estimates and variability	+	+	+	+	+	+	+	+	+	+
Total	5/11	5/11	5/11	5/11	3/11	5/11	5/11	5/11	5/11	5/11

### Jump Performance

Six studies analyzed jump performance with the Board Jump test [30], the Vertical Jump test [15], the Drop Jump test [13, 31] and the countermovement jump test [10, 18].

**Fig. 2:**
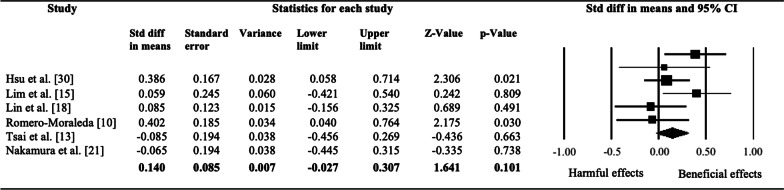
Effects of vibration foam roller intervention on jump performance in healthy adults. Values shown are effects sizes (standard mean differences) with 95% confidence intervals. The size of the plotted squares reflects the statistical weight of each study. The black diamond reflects the overall result

For this variable, three studies found an increase in jump performance with VFR but similar to other interventions such as FR and dynamic stretching (DS) [10, 18, 27]. Lim and Park [15] and Nakamura et al. [21] reported no benefits of VFR in jump performance, nor with FR, and Tsai et al. [13] found significant improvements with FR in jump performance but not with VFR. The results of the meta-analysis showed no significant effects between pre-and post-intervention with VFR (SMD = 0.14 [95% CI − 0.022 to 0.307]; *p* = 0.101; *I*^*2*^ = 1.08%) on jump performance. The relative weight of each study in the analysis varied between 13.85 and 36.84% (indicated by the size of the plotted box in Fig. 2).

### Agility

Three studies analyzed agility with the Figure-of-8 Hop test [31], the Edgren Side Step test [30] or the FITLIGHT test [18]. Agility was analyzed before and after an intervention with VFR. Lyu et al. [31] found a significant increase in agility after VFR with similar effects with and without dynamic contraction. Hsu et al. [30] reported an increase in agility after VFR with DS but similar to DS with FR without vibration and Lin et al. [18] showed an increase of the agility after VFR with DS but similar to the increase only with DS. For agility, the meta-analysis was not conducted due to the small number of studies analyzing this variable.

### Isokinetic Strength

**Fig. 3:**
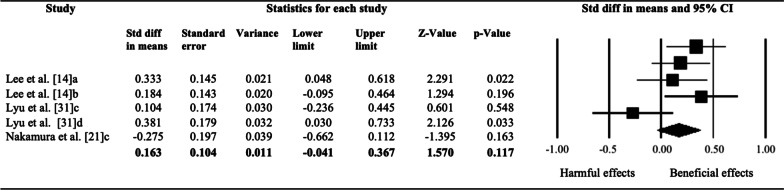
Effects of vibration foam roller intervention on isokinetic strength in healthy adults. Values shown are effects sizes (standard mean differences) with 95% confidence intervals. The size of the plotted squares reflects the statistical weight of each study. The black diamond reflects the overall result. a: Strength in quadriceps; b: strength in hamstrings; c: strength in plantar flexors; d: strength in dorsal flexors

Four studies analyzed the isokinetic strength, all of them with an isokinetic dynamometer. Lee et al. [14] and Chen et al. [19] tested the strength of the quadriceps and hamstrings with the dynamometer, assessing the knee joint, and Lyu et al. [31] and Nakamura et al. [21] performed the test on the ankle, analyzing the strength of the dorsal and plantar flexors. Lee et al. [14] found an increase in the strength of the quadriceps and hamstrings after VFR intervention, but similar to FR. Lyu et al. [31] reported an increase in the isokinetic strength of the ankle after VFR with DS and only after DS, with no significant differences between the two interventions, Nakamura et al. [21] reported no effects of VFR on strength and Chen et al. [19] analyzed the isokinetic strength of quadriceps and hamstrings but their design did not provide pre and post-test measurements so the meta-analysis could not be performed. The results of the meta-analysis reported no significant effects between pre-and post-intervention with VFR (SMD = 0.16 [95% CI − 0.041 to 0.367]; *p* = 0.117; *I*^*2*^ = 9.7%) on isokinetic strength performance. The relative weight of each study in the analysis varied between 15.3 and 21.39% (indicated by the size of the plotted box in Fig. 3).

### Recovery

The effects of VFR on recovery have been analyzed with different variables. Lai et al. [9] assessed the blood flow before and after an intervention with VFR and reported an increase of this variable, but similar to after FR. Chen et al. [19] analyzed the fatigue after training and recovery with VFR and DS, showing greater improvements in decreasing fatigue with this method in comparison with DS and SS combined with DS. Romero-Moraleda et al. [10] analyzed recovery in terms of perceived pain, and after an intervention with VFR this variable decreased significantly and more than with FR intervention. Similar to a previous review in this topic [7], the heterogeneity of the recovery variables made data consolidation and meta-analysis invalid.

## Discussion

This systematic review with meta-analysis presents a summary of the evidence available about the effects of VFR on jump performance, strength, agility and recovery. Results seem to indicate that short interventions with VFR do not have significant effects on jump performance and isokinetic strength. Recovery after exercise appears to improve with VFR interventions, in terms of pain, fatigue and blood flow, and agility seems to be enhanced with VFR interventions, but a meta-analysis of these variables was not conducted due to the heterogeneity of the measurements and the small number of investigations in these topics.

Foam rollers have been demonstrated to influence on the tissues involved, [2, 11]. With this intervention, the tissues are rolled and compressed and their mechanoreceptors are stimulated due to the pressure and the movement, producing changes in muscle and myofascial thixotropy, fascial hydration and blood flow [7, 11, 29–31]. All of these effects have been demonstrated to influence the sympathetic and parasympathetic systems by modulating the global pain systems and influencing the muscle tone and the stiffness [32, 33]. Recently, vibration has been added to the FR with the purpose of increasing the stimulation of the mechanoreceptors and enhancing the response of the tissues involved [8, 16, 20, 34]. Vibration was supposed to produce a more in-depth stimulation of the muscle and myofascia due to a greater contribution of the mechanoreceptors, specifically the interstitial type I and II receptors, which respond to a sustained pressure and modulate the sympathetic and parasympathetic activity [11, 20]. Nevertheless, the influence of this in-depth stimulation on variables of performance and recovery remains unclear.

Regarding jump performance, this review and meta-analysis showed no significant effects of VFR (Fig. 2). The modulation of the stiffness and the changes in the mechanical properties of the muscles rolled could produce an increase of the co-activation and contraction of the muscles involved, differently from interventions only with vibration [12]. Moreover, most investigations included in this systematic review and meta-analysis performed the intervention with VFR in all areas of the lower limb (anterior and posterior thigh and calf) and the low back, showing greater effects in comparison with interventions in one muscle, which could change the mechanical properties of the muscle [13, 18, 27]. A recent systematic review concluded the longer time spent with FR, the longer effects seemed to last [7], but to date no studies have assessed the effects of long-lasting interventions with FR on several muscles in comparison with shorter interventions.

Regarding agility, it seems to increase after interventions with VFR. Theoretically, the stimulation of the proprioceptors with VFR could increase the velocity of contraction and response [35, 36]. However, agility is influenced by the tone and the stiffness of the muscles involved [30, 39] and VFR has been shown to decrease these [3, 6, 37], which could be detrimental to agility test performance and could explain the conflicting results.

Conversely, the results of the meta-analysis show there is not enough evidence to assert that VFR has positive effects on isokinetic strength. Changes in tissue thixotropy, tone and stiffness could influence these results, but in addition the lower muscle activation reached with VFR could decrease the strength [8, 35, 36]. Previous studies with FR demonstrated that the strength could decrease after those interventions but maintaining the performance, due to the modulation of the tone and the changes in the mechanical properties of the muscles [32, 38, 39], but there is little evidence to support this theory with VFR.

The heterogeneity of the variables measured made the meta-analysis not possible for recovery. However, the results of the studies appear to support the idea that VFR enhances recovery after exercise, since the blood flow increased, and the perceived fatigue and pain decreased with VFR interventions. Specifically, the increase of blood flow is one of the main physiological explanations for the effects of VFR [29–31, 40] since it has been demonstrated to contribute to a better environment for muscle recovery, removing the inflammatory substances after exercise [10, 30, 41]. This increase of blood flow added to the changes in mechanical and thixotropic properties of the muscles involved and the modulation of the global pain system could explain the decrease of pain and fatigue reported [10, 19]. Similar to previous studies with FR [20, 37, 42, 43], the pressure applied during the intervention with VFR appears to stimulate and modulate the autonomic nervous system, influencing pain and fatigue perceived. However, there is not enough evidence to state that vibration increases these benefits.

The results of the present systematic review and meta-analysis provide evidence about the effects of VFR on performance and recovery; however, several limitations should be considered. First, the studies included contained limited sample sizes and had poor methodological quality according to the PEDro scale, especially with respect to blinding and concealment of allocation, probably due to the characteristics of the intervention with VFR  [44–49]. Second, there is little evidence analyzing the effects of VFR on performance and recovery, so the meta-analysis was conducted with few studies. Third, the lack of consistency in the duration of the interventions, the density of the devices and the frequency used for the vibration may explain the different findings. Future research should consider these limitations and perform randomized trials with higher levels of methodological quality, bigger sample sizes and similar interventions to provide more consistent results. Moreover, although there is some evidence to support the underlying physiological effects of VFR, they remain unclear and future research should explore this further.

## Conclusion

This systematic review with meta-analysis shows that VFR has a great potential to improve jump performance, agility, strength and recovery, but no significant results were found in these variables. Although the underlying physiological effects of VFR are not fully understood, the potential of this method invites wider research in this field.

## Data Availability

Data sharing is not applicable to this article as no datasets were generated or analyzed during the current study.
